# Supplementary material for: Allosteric regulation and crystallographic fragment screening of SARS-CoV-2 NSP15 endoribonuclease
Source: Nucleic Acids Res. 2023 Apr 28;51(10):5255–70. doi: 10.1093/nar/gkad314 (PMC10250223; doi:10.1093/nar/gkad314)
Supplement: gkad314_Supplemental_Files [file gkad314_supplemental_files.zip › Supplementary datasets.zip!/81/Refine_9/01-BUSTER/Cycle-1/shell.01/R_Rfree.html]

# Rxpct factors in resolution bins at cycle 0


|  |  |  |  |  |  |  |  |  |
| --- | --- | --- | --- | --- | --- | --- | --- | --- |
|  | **<d> (Å)**  Rxpct\_Work | Rxpct\_Free | Rxpct\_All | |  |  |  |  |
|  | 15.0454 | 0.2858 | 0.2245 | 0.2835 |  |
|  | 8.6865 | 0.1933 | 0.2379 | 0.1959 |  |
|  | 6.7285 | 0.2599 | 0.2240 | 0.2578 |  |
|  | 5.6866 | 0.2786 | 0.2567 | 0.2779 |  |
|  | 5.0151 | 0.2067 | 0.2101 | 0.2069 |  |
|  | 4.5364 | 0.1883 | 0.1897 | 0.1883 |  |
|  | 4.1728 | 0.2070 | 0.1813 | 0.2056 |  |
|  | 3.8847 | 0.2414 | 0.2426 | 0.2415 |  |
|  | 3.6491 | 0.2460 | 0.2459 | 0.2460 |  |
|  | 3.4517 | 0.2514 | 0.2222 | 0.2500 |  |
|  | 3.2832 | 0.2481 | 0.2645 | 0.2491 |  |
|  | 3.1372 | 0.2253 | 0.2343 | 0.2257 |  |
|  | 3.0091 | 0.2250 | 0.2367 | 0.2254 |  |
|  | 2.8955 | 0.2203 | 0.2123 | 0.2199 |  |
|  | 2.7939 | 0.2210 | 0.1948 | 0.2198 |  |
|  | 2.7022 | 0.2173 | 0.2287 | 0.2179 |  |
|  | 2.6191 | 0.2186 | 0.2326 | 0.2194 |  |
|  | 2.5431 | 0.2084 | 0.1985 | 0.2078 |  |
|  | 2.4735 | 0.2054 | 0.2005 | 0.2052 |  |
|  | 2.4092 | 0.2172 | 0.1936 | 0.2162 |  |
|  | **Overall** | 0.2289 | 0.2221 | 0.2285 |  |

# Rxpct factors in resolution bins at cycle 1


|  |  |  |  |  |  |  |  |  |
| --- | --- | --- | --- | --- | --- | --- | --- | --- |
|  | **<d> (Å)**  Rxpct\_Work | Rxpct\_Free | Rxpct\_All | |  |  |  |  |
|  | 15.0454 | 0.2849 | 0.2208 | 0.2825 |  |
|  | 8.6865 | 0.1928 | 0.2385 | 0.1954 |  |
|  | 6.7285 | 0.2567 | 0.2219 | 0.2547 |  |
|  | 5.6866 | 0.2747 | 0.2559 | 0.2741 |  |
|  | 5.0151 | 0.2051 | 0.2070 | 0.2053 |  |
|  | 4.5364 | 0.1891 | 0.1870 | 0.1890 |  |
|  | 4.1728 | 0.2072 | 0.1806 | 0.2058 |  |
|  | 3.8847 | 0.2428 | 0.2432 | 0.2428 |  |
|  | 3.6491 | 0.2463 | 0.2460 | 0.2463 |  |
|  | 3.4517 | 0.2499 | 0.2169 | 0.2483 |  |
|  | 3.2832 | 0.2462 | 0.2608 | 0.2471 |  |
|  | 3.1372 | 0.2246 | 0.2335 | 0.2250 |  |
|  | 3.0091 | 0.2231 | 0.2349 | 0.2236 |  |
|  | 2.8955 | 0.2199 | 0.2195 | 0.2199 |  |
|  | 2.7939 | 0.2216 | 0.2002 | 0.2206 |  |
|  | 2.7022 | 0.2186 | 0.2334 | 0.2194 |  |
|  | 2.6191 | 0.2189 | 0.2395 | 0.2201 |  |
|  | 2.5431 | 0.2119 | 0.2031 | 0.2113 |  |
|  | 2.4735 | 0.2117 | 0.1973 | 0.2110 |  |
|  | 2.4092 | 0.2199 | 0.1981 | 0.2189 |  |
|  | **Overall** | 0.2285 | 0.2215 | 0.2281 |  |

# Rxpct factors in resolution bins at cycle 2


|  |  |  |  |  |  |  |  |  |
| --- | --- | --- | --- | --- | --- | --- | --- | --- |
|  | **<d> (Å)**  Rxpct\_Work | Rxpct\_Free | Rxpct\_All | |  |  |  |  |
|  | 15.0454 | 0.2853 | 0.2177 | 0.2827 |  |
|  | 8.6865 | 0.1951 | 0.2434 | 0.1980 |  |
|  | 6.7285 | 0.2627 | 0.2346 | 0.2611 |  |
|  | 5.6866 | 0.2804 | 0.2634 | 0.2798 |  |
|  | 5.0151 | 0.2085 | 0.2088 | 0.2085 |  |
|  | 4.5364 | 0.1884 | 0.1878 | 0.1884 |  |
|  | 4.1728 | 0.2063 | 0.1811 | 0.2050 |  |
|  | 3.8847 | 0.2407 | 0.2398 | 0.2406 |  |
|  | 3.6491 | 0.2449 | 0.2470 | 0.2450 |  |
|  | 3.4517 | 0.2500 | 0.2187 | 0.2485 |  |
|  | 3.2832 | 0.2473 | 0.2593 | 0.2480 |  |
|  | 3.1372 | 0.2254 | 0.2340 | 0.2258 |  |
|  | 3.0091 | 0.2230 | 0.2346 | 0.2234 |  |
|  | 2.8955 | 0.2182 | 0.2165 | 0.2181 |  |
|  | 2.7939 | 0.2195 | 0.1994 | 0.2186 |  |
|  | 2.7022 | 0.2163 | 0.2305 | 0.2171 |  |
|  | 2.6191 | 0.2144 | 0.2321 | 0.2154 |  |
|  | 2.5431 | 0.2049 | 0.1969 | 0.2044 |  |
|  | 2.4735 | 0.2039 | 0.1861 | 0.2030 |  |
|  | 2.4092 | 0.2132 | 0.1981 | 0.2125 |  |
|  | **Overall** | 0.2286 | 0.2222 | 0.2283 |  |

# Rxpct factors in resolution bins at cycle 3


|  |  |  |  |  |  |  |  |  |
| --- | --- | --- | --- | --- | --- | --- | --- | --- |
|  | **<d> (Å)**  Rxpct\_Work | Rxpct\_Free | Rxpct\_All | |  |  |  |  |
|  | 15.0454 | 0.2862 | 0.2203 | 0.2837 |  |
|  | 8.6865 | 0.1979 | 0.2404 | 0.2004 |  |
|  | 6.7285 | 0.2617 | 0.2295 | 0.2598 |  |
|  | 5.6866 | 0.2750 | 0.2637 | 0.2746 |  |
|  | 5.0151 | 0.2051 | 0.2033 | 0.2049 |  |
|  | 4.5364 | 0.1844 | 0.1889 | 0.1846 |  |
|  | 4.1728 | 0.2037 | 0.1782 | 0.2023 |  |
|  | 3.8847 | 0.2355 | 0.2295 | 0.2352 |  |
|  | 3.6491 | 0.2435 | 0.2360 | 0.2431 |  |
|  | 3.4517 | 0.2449 | 0.2243 | 0.2439 |  |
|  | 3.2832 | 0.2417 | 0.2548 | 0.2425 |  |
|  | 3.1372 | 0.2191 | 0.2263 | 0.2195 |  |
|  | 3.0091 | 0.2189 | 0.2226 | 0.2191 |  |
|  | 2.8955 | 0.2100 | 0.2060 | 0.2098 |  |
|  | 2.7939 | 0.2124 | 0.1858 | 0.2112 |  |
|  | 2.7022 | 0.2087 | 0.2245 | 0.2095 |  |
|  | 2.6191 | 0.2092 | 0.2349 | 0.2108 |  |
|  | 2.5431 | 0.2034 | 0.2009 | 0.2033 |  |
|  | 2.4735 | 0.2005 | 0.1942 | 0.2002 |  |
|  | 2.4092 | 0.2125 | 0.1913 | 0.2116 |  |
|  | **Overall** | 0.2251 | 0.2182 | 0.2247 |  |
